# Nursing Personnel in the Era of Personalized Healthcare in Clinical Practice

**DOI:** 10.3390/jpm10030056

**Published:** 2020-06-29

**Authors:** Marios Spanakis, Athina E. Patelarou, Evridiki Patelarou

**Affiliations:** 1 Computational BioMedicine Laboratory, Institute of Computer Science, Foundation for Research and Technology—Hellas (FORTH), Heraklion, GR-70013 Crete, Greece; 2 Department of Nursing, Faculty of Health Sciences, Hellenic Mediterranean University, Heraklion, GR-71004 Crete, Greece; apatelarou@hmu.gr (A.E.P.); epatelarou@hmu.gr (E.P.)

**Keywords:** personalized medicine, stratified medicine, precision health, personalized nursing care, evidence-based practice

## Abstract

Personalized, stratified, or precision medicine (PM) introduces a new era in healthcare that tries to identify and predict optimum treatment outcomes for a patient or a cohort. It also introduces new scientific terminologies regarding therapeutic approaches and the need of their adoption from healthcare providers. Till today, evidence-based practice (EBP) was focusing on population averages and their variances among cohorts for clinical values that are essential for optimizing healthcare outcome. It can be stated that EBP and PM are complementary approaches for a modern healthcare system. Healthcare providers through EBP often see the forest (population averages) but miss the trees (individual patients), whereas utilization of PM may not see the forest for the trees. Nursing personnel (NP) play an important role in modern healthcare since they are consulting, educating, and providing care to patients whose needs often needs to be individualized (personalized nursing care, PNC). Based on the clinical issues earlier addressed from clinical pharmacology, EBP, and now encompassed in PM, this review tries to describe the challenges that NP have to face in order to meet the requisites of the new era in healthcare. It presents the demands that should be met for upgrading the provided education and expertise of NP toward an updated role in a modern healthcare system.

## 1. Introduction

The achievements in health-related scientific disciplines continuously gain attention among healthcare providers as well as among academia and industry [1]. Their main goal is the implementation of new approaches in healthcare, oriented as personalized and stratified medicine which, nowadays, in order to follow a unified scientific approach and terminology, are often referred to as precision medicine (PM) [2]. These advancements represent new holistic methods in healthcare aiming to exploit and extrapolate the generated knowledge from bench research to the bedside at a clinical level towards the optimization of provided therapy regarding outcome and reduced costs of a modern healthcare system with advanced services for each patient. All these innovative systemic medicine approaches try to combine state-of-the-art knowledge deriving from biology, pharmacology, chemistry, physics, and other scientific fields, in order to develop novel therapeutic methods for various diseases and medical conditions [3]. As a result, over the previous years, new scientific fields have emerged in PM toward a new era in medicine, and it is shifting from reactive or one-size-fits all scenarios, toward participatory, preventive, predictive, and personalized (P4) methods [4,5]. Till today, PM initiatives have been launched in many parts of the world, especially in the U.S. and Europe and they introduce new terminologies that are continuously utilized in clinical settings [6,7]. 

**Figure 1:**
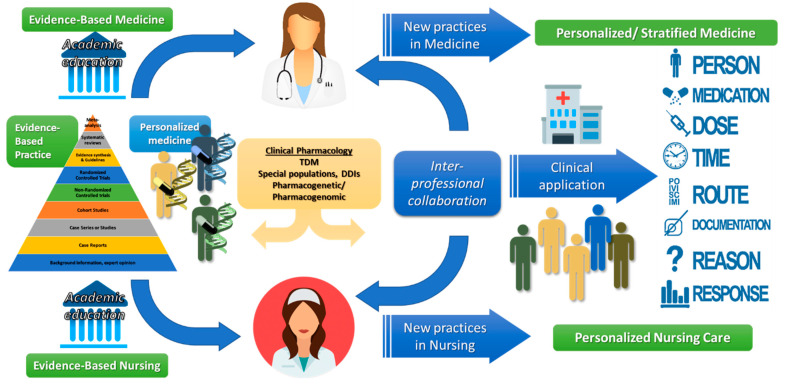
The role of nursing personnel (NP) in utilizing knowledge and interpreting instructions derived from treating physicians in clinical settings toward personalized medicine and nursing care.

This modern era is also a new challenge for medical personnel and healthcare providers, and it is crucial for them to advance their expertise on these fields through their continuous education and awareness [8]. Nursing personnel (NP), as members of a modern healthcare system and the largest workforce of healthcare providers, have a crucial role in order to sustain the goals of PM for hospital clinics, smaller healthcare units, or even in home care [9]. Their duties are mainly related with care for a small patient cohort of a clinic, or a patient who is health cared at home (i.e., older people). NP should also meet up to the challenges posed from modern healthcare systems and thus enhance their collaboration with treating physicians to implement advanced clinical practices as derived from PM (Figure 1). This review aims to provide a brief description of how clinical pharmacology and pharmacogenomics/pharmacogenetics as well as, evidence-based practice (EBP) basically are exploited today through PM and more importantly, why all this new knowledge should be provided to and utilized by NP, as members of a modern healthcare system.

### 1.1. Clinical Pharmacology, Therapeutic Drug Monitoring (TDM), Special Population Groups, and Pharmacogenetics/Pharmacogenomics

Although PM is considered the next era in medicine, the basic principles were founded and previously utilized in clinical pharmacology and in the fitting of medical treatment to the individual characteristics of each patient [10,11]. Clinical pharmacology incorporates basic (e.g., molecular) and applied pharmacology (e.g., pharmacokinetics (PK), pharmacodynamics, (PD)) from preclinical drug research and development (R&D) up to all phases of clinical trials and finally to clinical application [10,11,12,13]. Typical examples previously addressed through clinical pharmacology and nowadays targeted in PM are as follows: (i) therapeutic drug monitoring, (ii) pharmacotherapy administration in special population groups, and (iii) utilization in clinical practice of pharmacogenetic/pharmacogenomic patient data [11]. In all these cases, the understanding of the inter- and intra-subject variabilities regarding pharmacological response should be considered in order that the general principle of “the right drug, at the right dose, at the right route, at the right time, with the right documentation, for the right reason, at the right patient” is followed (Figure 1) [14,15].

Therapeutic drug monitoring (TDM) is applied for drugs with narrow therapeutic index, inter-subject variability in PK parameters, correlation between plasma concentrations and pharmacological outcome, and available validated bioanalytical methods to quantifiy drug concentrations. Well-known examples of drugs undergoing TDM are digoxin, lithium, cyclosporine, phenytoin, tacrolimus, sirolimus, warfarin, antibiotics, and chemotherapy agents. The importance of clinical interpretation is based on the accuracy in the established TDM protocol including parameters such as time of blood sampling, handling of samples, validation of bioanalytical method, PK analysis, possible drug interactions, and personalization parameters for a patient (renal function, comorbidities, etc.). In all these cases, it is essential that NP fully grasp the requirements that should be met regarding TDM practices and processes as they usually perform the blood sampling for the analysis and administer the prescribed dosing scheme [15,16].

Special population groups (i.e., the elderly, those with renal failure, pregnant women, and neonates) experience changes due to disease progression or modulation of physiology (intra-subject variability), which can modulate the PK parameters for an administered drug and thus alter the pharmacological outcome [17,18,19,20]. Data for special populations and drug administration are continuously adopted in clinical settings and this knowledge should be acquired as best as possible by NP (and all medical personnel), which will help them to fully understand the importance of individualizing the provision of healthcare under such cases. For example, in kidney disease or liver dysfunction, the alteration of PK processes and parameters, such as drug metabolism and clearance, can alter the clinical outcome due to higher concentrations for drugs that are either metabolized through liver enzymes or cleared through kidneys [21,22]. 

Pharmacogenetic and pharmacogenomic variations are considered an important source of variability in drug response that potentially modulates drug efficacy among patients or the incidence of adverse drug reactions (ADRs) and requires additional considerations in pharmacotherapy administration [23,24,25]. Single-nucleotide polymorphisms (SNPs) occur when a single nucleotide in the individual’s genome differs between paired chromosomes resulting in two alleles. In cases where SNPs are related with a drug’s pharmacological mechanism, they can affect how patients respond to several medications. SNPs today represent a key concept for PM, whereas their role is well known in clinical pharmacology [26]. These inter-subject differences can be important for ADR incidences or reduced drug response. Maybe the most known population variability in clinical pharmacology where the phenotype plays important role—especially in PK—is regarding liver metabolizing enzymes of phase I oxidation reactions (i.e., cytochrome P450, CYPs) or phase II conjugation reactions (i.e., UDP-glucuronosyltransferase, UDPs) for metabolic catalysis of drugs prior to their elimination [27,28]. CYP2D6 is an outstanding example with phenotypes stratifying patients in distinct groups (poor, intermediate, extensive, and ultra-rapid metabolizers) and administration of the same dose can result in different drug concentrations depending on which phenotype group a patient is stratified into [29]. Apart of CYP2D6, other CYP polymorphisms with clinical impact are for CYP2C9, CYP2C19, CYP2D6 and CYP3A4/5 enzymes. Respectively, similar inter-subject variability for phase II reactions can be found for thiopurine methyltransferase (TPMT), UDP-glucuronosyltransferase (UGT1A1), N-acetyltransferase 2 (NAT2), and glutathione S-transferase (GST) [27,28,30]. Genetic and genomic knowledge can also assist in the development of targeted therapies as it is aimed in PM [24]. One of the most well-known examples in this case is warfarin which is based on pharmacogenomic information for its target (vitamin K epoxide reductase, VKORC1) and its primary metabolic enzyme (CYP2C9) requires warfarin to undergo TDM [31]. Moreover, in the era of PM in oncology, pharmacogenomics nowadays provides essential information for therapy outcome such as in cases of Kirsten rat sarcoma oncogene or KRAS (cetuximab/panitumumab), v-raf murine sarcoma viral oncogene homolog B or BRAF (vemurafenib), epidermal growth factor receptor or EGFR (gefitinib, erlotinib), tyrosine-protein kinase KIT CD117 (cluster of differentiation 117) or c-KIT (imatinib) and microtubule-associated protein-like 4-anaplastic aymphoma Kinase or EML4-ALK (crizotinib) [32,33,34,35,36]. In addition, harnessing the capabilities of modeling and simulation (M&S) for evidence synthesis from inferential methods, more and more scientific approaches try to implement PK/PD M&S regarding administration of drugs in special population groups with genomic/genetic variances, for several drug administration scenarios such as drugs that require TDM or show intra-/inter-subject variabilities in PK/PD profiles [37,38,39,40,41,42]. Especially for new drugs, these approaches are increasingly expected by the regulatory body for labeling decisions [39,43]. 

NP’s role is usually related with pharmacotherapy administration and since these data are adopted in clinical settings, it is essential for this knowledge to be acquired as best as possible by NP in order that dosing errors and the risk of toxicity may be avoided in cases where patients belong to a special population group, and moreover NP should be capable of utilizing different procedures in such cases [17,44]. NP should consider that patients—even if diagnosed with same disease(s)—may represent different cases. Differences due to changes in physiology from disease progression, disease genomic background, or underlying polymorphisms can result in different pharmacological and clinical outcomes between patients with the same disease and similar dose administration of the same drug. In this respect, the attending NP should be able to recognize and report potential differences in the observed clinical outcome and moreover should be capable of associating any potential intra- and/or inter-subject variability in genetic/genomic variances among subjects. NP convey information from patients to physicians and vice versa as they perform many healthcare procedures such as drug administration, blood sampling, preparation for examination, monitoring, recording, and updating patient’s health status. Thus, NP, in order to be able to provide advanced clinical practice in modern healthcare system should fully acquire and utilize all this knowledge and integrate these new fields in continuing professional development regarding rationality, effectiveness, and safety of therapy administration in patients. 

### 1.2. Personalized Nursing Care: What Does the New Nursing Role Entail for the Goals of Evidence-Based Practice (EBP) and PM?

Evidence-based medicine (EBM) and practice (EBP), as a wider term, can be described as the “conscientious, explicit, and judicious use of current best evidence in making decisions about the care of individual patients” [45,46,47]. The main goal of EBP is the optimization of healthcare based on integration of clinical expertise, patients’ values, and well-thought-out quality of scientific evidence that is followed in a clinical setting regarding a disease or a condition by all the medical professionals, including NP [47]. The quality of evidence is based on the availability of scientific data and often described as the EBM triangle that demonstrates the build-up of evidence from observational studies up to meta-analysis data for clinical appraisal [48,49]. Generally, in EBM/EBP, data are collected from large cohorts and clinical studies to estimate mean values and variances among population groups (i.e., demographics, physiology, pathology, -omics, etc.) for clinical values that are essential for optimizing healthcare outcome [44,46,50,51]. Although EBM/EBP represent the highest quality of evidence regarding a therapeutic intervention for a population, they may not apply for a specific individual with values outside the average distribution or for clinical data with low external validity [51,52,53]. 

**Figure 2:**
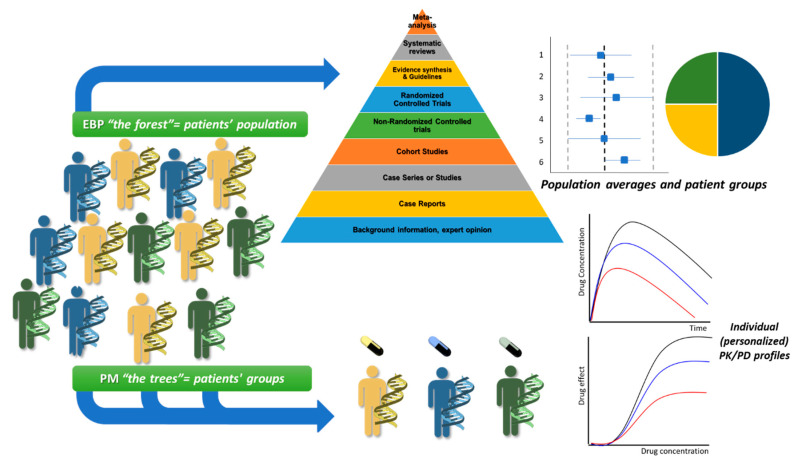
Evidence-based practice (EBP) and precision medicine (PM) represent complementary approaches in a modern healthcare system.

PM on the other hand is focusing on factors that explain the individual variability and thus seem to address the issues and limitations of EBP. In PM, multiscale data deriving from clinical profiles for each patient along with -omics data (genomic, proteomic, pharmacogenomic, etc.) are integrated in order to stratify each patient into fitting cohorts with similar characteristics [54,55,56]. The main goal of personalized medicine initially was described as to provide the best therapy per person or optimized for a group of patients with similar characteristics (stratified medicine) [56,57]. Limitations in PM are also present and mostly related with the data size analysis for each patient, but the advancements in computational science today try to address them [58,59]. Nowadays, PM approaches are continuously developed for chronic and complex diseases (i.e., cardiovascular, diabetes, arthritis, kidney disease) and mainly for cancer [60,61,62,63,64,65,66,67]. Their exploitation is continuously providing (i) advanced innovative tools in academia; (ii) data for the pharmaceutical industry to predict the most promising compound during R&D; and (iii) novel aspects in medical society as decision-making tools for optimum treatment response, which are applied in clinical practice [68,69]. It can be proposed that although EBP seems to oppose PM, they are actually mutually complementary both in their goals but also in their limitations [45,50]. When it comes to optimization of provided healthcare, EBP and the population approaches “sees the forest but miss the trees”, whereas, PM by focusing on each patient, sometimes “can’t see the forest for the trees” (Figure 2). 

NP, as they are in direct contact with patients, represent a key factor for the goals of both EBP and PM. Their responsibilities to provide, monitor, educate, and advice individual patients and their families regarding administered medications and treatments aim toward what is today described as personalized nursing care (PNC). PNC although a term not yet clearly defined in nursing comparing to other disciplines, including medicine and computer sciences, emerged in the nursing literature early in 1960 and describes the need of provision of care intended for a particular patient at a particular time point [70]. PNC entails the uniqueness of a human being, his/her distinctiveness in character, and own individual needs and prerequisites inter-professional collaboration between nurses, physicians, computer scientists, pharmacists, other bio-scientists, and social workers. PNC promotes user-driven, self-health-seeking behaviors through in-depth participation, self-control, and self-promotion, which further relate to patient satisfaction and improved health outcomes [71,72]. PNC includes an individual’s genetic and genomic information to make decisions about his/her care, in line with personal, health, and environmental factors [73]. This is translated into the need for interpretation and clinical use of novel and personalized information including genetic testing, patient advocacy, and support throughout testing, anticipating results and treatment, ongoing chronic monitoring, and support for patient decision-making. 

In the context of PNC, it is important for NP to gain competencies in order to be able to follow the instructions given to them and, thus, be familiar with terminologies that PM uses in today’s clinical practice (Table 1) [9,74,75,76]. Indeed, nurses providing PNC should have a good level of understanding and ability to explain genetic and genomic tests; to be able to navigate the ethical, legal, and social issues involved in genetic and genomic testing; and to be aware of the referral base for genetic counseling, clinical trials, or specialty care.

**Figure 3:**
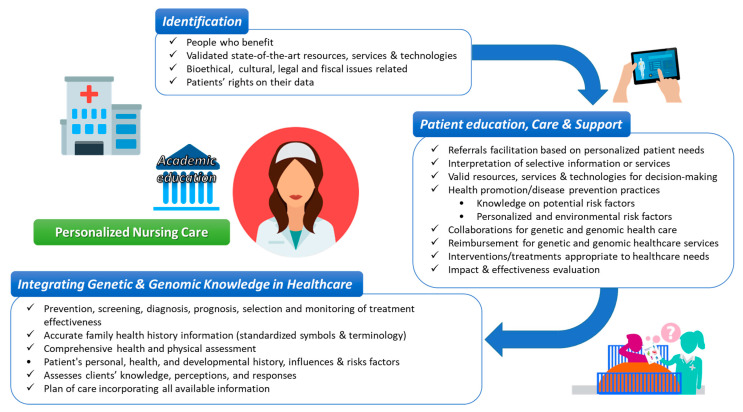
Personalized nursing care (PNC) in the context of PM that utilizes genomic competencies from NP.

Implications of PM for PNC relate to all stages of care: i.e., prediagnosis, diagnostics, treatment, and prognosis stages (Figure 3). NP play a key role as they interact and provide information and guidance to patients prior genetic or other testing. At the “prediagnosis stage”, NP are expected to assess the clinical risk factors, perform screening as appropriate, and explain the results of genetic risk testing to the family. NP need to discuss patient values and priorities, provide all information needed in order to enhance decision-making, and provide support for family notification and testing [77]. NP need to be able to explain and discuss the implications of genetic testing and other risk testing with patients and their family members in an informed manner. The “diagnostics stage” involves the understanding of and ability to explain the tests, their validity, and the meaning of associated results to the patient. Providing the level of information that the patient can handle and as much information as possible while facilitating unbiased support are essential practices and skills for the nurse at this stage [78]. The “treatment stage” involves the identification of patients for whom any -omics or genetic testing platforms are appropriate for guidance in treatment considerations or prognostication, the discussion of recommended therapies, and the use of clinical decision-support tools to integrate personalized approaches and patient data (biomarker, patient-reported data, clinical data, etc.) into patient treatment plan discussions. Finally, the “prognosis stage” entails ongoing monitoring and management, which translates to supporting the patient in determining and following through on family implications for genetic test results and to providing psychosocial support for the patient. NP may need to assist patients with management of care between specialists and primary healthcare services if required. This may involve communication with patients to ensure an accurate understanding of their disease status and plan of care, as well as coordinated record sharing and/or communication with other healthcare providers. 

### 1.3. Milestones for NP toward the Requirements of A Modern Healthcare System

**Figure 4:**
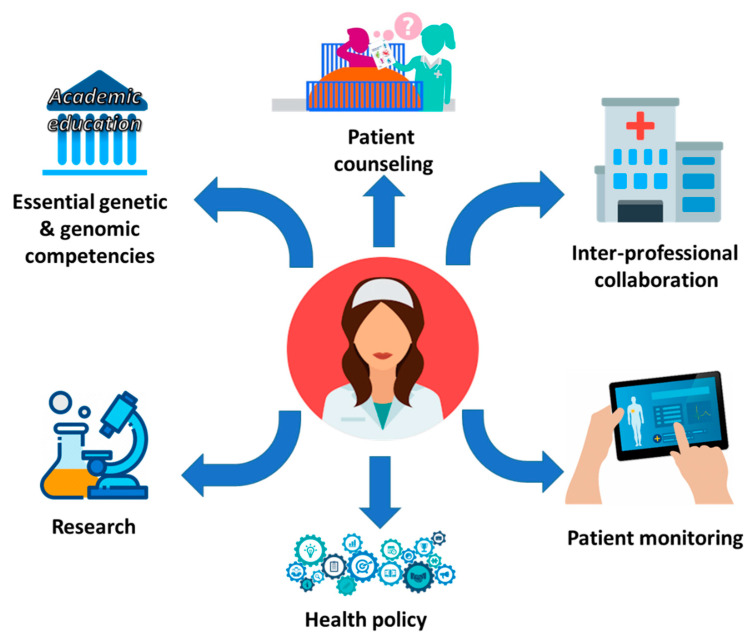
NP’s competencies regarding PM knowledge will give them an updated role in a modern healthcare system.

PNC in PM involves the interpretation and clinical use of -omics information toward patient advocacy and support throughout diagnosis, treatment adherence and compliance, and follow-up monitoring [79]. In this respect, NP have to stay up to date with the changes generated from the realization in clinical practice of PM and moreover, integrate relative content for their continuous education [80]. This will provide NP the means to adopt state-of-the-art approaches to the bedside, essentially contributing to symptom assessment, prevention, management, and disease treatment toward optimal healthcare in their patients (e.g., a special population of elder patients) [81]. This realization in clinical settings requires the overcoming of characteristic milestones that will allow NP to look beyond the current challenges toward their new role in a modern healthcare system [80]. These milestones are related with education of NP and how this knowledge will be utilized in clinical settings both with treating physicians (inter-professional collaborations) and patients (patient awareness) so that NP can actively participate in health policy as well as conduct their own research (Figure 4). 

The education of NP should be adjusted to the requirements of modern medicine. The adoption of educational strategies in nursing that promote evidence and innovations and motivate undergraduate and postgraduate students to get involved with EBP implementation can be considered as an a priori necessity [82]. EBP principles will allow NP to better grasp the subjects of patient cohorts, mean values and variances among populations, or individual characteristics that are subsequently exploited in PM. Thus, it is a necessity to integrate genomics/genetics in nursing curricula at the undergraduate level as part of the basic training [83,84]. In this way, NP will have the opportunity to increase the level of genomics competence and knowledge at an early stage. More importantly, this knowledge can be exploited, so that NP will delve further into such subjects through postgraduate programs and Ph.D. research projects related with precision health and PNC [84]. Clinical research in combination with clinical expertise and patients’ preferences are considered to be the three components of EBP as it was first described 25 years back [85]. Today, after all these years, it is evident that NP scientists must develop adequate competencies and knowledge so as to promote research and enhance new methods of personalized care, prevention, and management across the lifespan [80,85]. A final step for dissemination among NP of the knowledge gained and adopted in clinical settings regarding PM is the continuous provision of information and educational resources for NP (Table 2). Today, associations and alliances at the national and international level offer seminars, workshops, courses, and free online resources regarding genomics/genetics. The American Association of Nurses has identified essential genetic and genomic competencies for individuals prepared at the graduate level in nursing including, but not limited, to advanced practice registered nurses (APRNs), clinical nurse leaders, nurse educators, nurse administrators, and nurse scientists. The experts found thirty-eight competencies focusing on risk assessment and interpretation; genetic education; counseling; testing and result interpretation; clinical management; ethical, legal, and social implications; professional role; leadership; and research [86,87]. 

A successful update in skills and knowledge of NP regarding PM goals will introduce new potentials in collaboration with other healthcare providers (treating physicians, clinical pharmacists, etc.). NP competencies and standards of practice in order to incorporate genetics and genomics into all clinical and non-clinical nursing roles have already been described, which set clear roles for the members of multidisciplinary healthcare teams in order to avoid future conflicts and identify common areas for collaboration and interaction among the healthcare providers for every health domain: acute care, long-term care, community nursing, public health nursing [80]. This positive impact of personalized and precision care in health safety promotion will raise public awareness toward improved and effective NP–patient communication. 

Cultivating a new NP–patient interaction and communication in the context of embracing genetics/genomics as a novel method that does not undermine patient’s right and is based on informed consent, and voluntary action will further disseminate the evidence that PM interventions can be considered a cost-effective method that improves healthcare quality and promotes well-being [84,86,87]. This approach will lead to a total implementation of PNC (data collection through accurate family health histories, risk factors, etc.). Already available literature addresses the importance of decision making in the era of precision health regarding accurate family history risk factors as well as patient needs and preferences that are encompassed in electronic health records [88,89]. 

Informational needs are strongly associated with organizational culture and management and, thus, they are of main importance for the EBP embracement in clinical practice [90]. There is evidence that stresses the need for clinical mentors who will reinforce the effort toward advancing PNC through innovation and implementation and limit the resistance to change [91,92]. Leadership and organizational culture have been strongly associated with quality care [93]. On the other hand, promotion of NP’s contribution to the field of genomics/genetics in order to participate in PM programs and conferences will add new professionals among international scientific networks in medical fields and the efforts to improve health outcomes and to implement innovations. In this respect, inspiring NP through nursing leaders and mentors and cultivating a spirit of inquiry as a first step for evidence-based practice implementation but also fostering nursing leaders’ role through further education in the field of PM will advance the role of NP not only in healthcare policy decisions but also in the clinical settings. 

Genomics is the backbone for future medical and nursing management of the disease. As a result, not only NP, but healthcare staff, in general, need to be upskilled to recognize the benefits and implications of genomic medicine. Even today, regulatory bodies (Food and Drug Administration, FDA and European Medicines Agency, EMA) demonstrate their commitment to accelerating PM realization and are continuously working to ensure the accuracy of the applied methods [94,95]. Hence, in collaboration with academia and along with medical associations, they could set some basic standards regarding the prerequisite knowledge that members of interdisciplinary medical teams should have regarding PM goals at a clinical level. Health professionals’ immersion in -omics must begin even from the undergraduate level after a medical and nursing curriculum reform. Genetics as current health professionals’ core competence should be embedded in each level of training, while NP, pharmacists, and physicians should be encouraged to receive additional training and continuing education in the field of -omics and informatics focusing on biomedical, translational, and nursing informatics [96].

## 2. Conclusions

Healthcare aimed at PM requires NP to be knowledgeable and competent in their understanding, synthesis, and application of these advances in science. Nursing education and continuing education, clinical decision support, and health systems changes will be necessary to provide personalized multidisciplinary care to patients, in which nurses play a key role. This will require both capacity building within the existing workforce and a new approach to the education of future generations. Potential ways forward for education and training include an extensive review of all nursing curricula and integration of genetics, genomics, mathematics, statistics, ethics, and computer/information and communications technology (ICTs) sciences at all levels of nursing education.

## Figures and Tables

**Table 1:** A brief list of basic terminologies that NP should be aware of since they are often used in clinical practice of PM.

Terminology (/Synonyms)	Brief Description
Personalized/stratified/precision & medicine/pharmacotherapy/healthcare	The use of an individual’s genetic and epigenetic information to tailor drug therapy or preventive care. It aims to stratify patients into similar cohorts with medical interventions to be customized based on individual/cohort characteristics.
Personal genomic profile	Sequencing, analysis, and interpretation of the genome for an individual (patient or citizen). It includes techniques for single-nucleotide polymorphism (SNP) analysis, partial, or full-genome sequencing. It is used to analyze trait expression, disease risk assessment, and ancestry.
Biomarker	A biological characteristic that is objectively measured and evaluated as an indicator or normal or pathological processes or used as response to a therapeutic intervention.
Genetic testing	Detection of specific alleles, mutations, genotypes, or karyotypes that are associated with heritable traits, diseases, or predispositions to disease for the individual or their descendants.
Genome-wide association studies (GWASs)	Genome screens of unrelated individuals and appropriately matched controls or parent–affected child trios to establish whether any genetic variant, i.e., any SNP is associated with a trait
Genomics	The full genetic complement of an organism that is sequenced, assembled, and analyzed regarding its structure and function.
Genomic analysis/profiling	Recognition, measurement, or comparison of genomic features (i.e., DNA sequences, gene expression or regulation, and functional element annotation) at a genomic scale.
Metabolomics	Systematic identification and quantification of the small-molecule metabolic products (the metabolome) of a biological system (cell, tissue, organ, biological fluid, or organism) at a specific point in time.
Molecular diagnosis	Identification of genomic variants to enable detection, diagnosis, subclassification, prognosis, and monitoring response to therapy.
Proteomics	Study of proteome or the techniques used to determine the entire set of proteins for an organism or a system.
Transcriptomics	The screening or analysis of the complete set of RNA (coding and non-coding) that is produced by the genome (from one or more cells) under specific circumstances using high-throughput methods.
Pharmacogenomics	The study of how genetic variation influences responses to drugs.
Pharmacogenetics	How an individual can benefit from specific drugs due to inter-subject genetic variation that affects their response to drugs/pharmaceuticals and other xenobiotics, both therapeutically and in terms of adverse effects.
Pharmacometrics	Implementation of model-based approaches of biology, physiology, pharmacology, and disease that aim to describe and quantify interactions among drugs (or other chemicals) and patients such as therapeutic response or adverse effects.
Single-nucleotide polymorphism	A DNA sequence variation occurring when a single nucleotide in the genome (or other shared sequence) differs between members of a species or paired chromosomes in an individual.

**Table 2:** Web sources regarding genetic/genomic information for healthcare providers and public.

Title	Link	Description	Target Group
Centers for Disease Control and Prevention National Public Health Genomics	https://www.cdc.gov/genomics/; https://www.cdc.gov/genomics/translation/GAPPNet/index.htm/	General Genetic/Genomic Resources/innovative public health genomics programs	Health Professionals, Scientists, Public
International Society of Nurses in Genetics	https://www.isong.org/page-1325075	Webinars, annual conference	Nurses
National Human Genome Research Institute	https://www.genome.gov/	Educational resources, research training, professional development programs	Health Professionals, Scientists, Public
Global Alliance for Genomics and Health	https://www.ga4gh.org/	Education resources, toolkit	Health Professionals, Scientists, Public
Global Genetics and Genomics Community	https://www.genomicscases.net/en/	Educational resources	Health Professionals, Scientists, Public
Genetics Education Program for Nurses (GEPN), Cincinnati Children’s	https://www.cincinnatichildrens.org/education/clinical/nursing/genetics	Free modules, audio-slide content	Nurses
Health Education England-Genomics Education Programme	https://www.genomicseducation.hee.nhs.uk/education/?swoof=1&product_cat=online-courses	Educational resources and online courses, Master’s degree	Health Professionals, Scientists
phgFoundation (Public Health Genomics Foundation), University of Cambridge	https://www.phgfoundation.org/	Bespoke training, training placements, internship opportunities	Public, post-graduate students, trainees and visiting scholars
The European Society of Human Genetics (ESHG)	https://www.eshg.org/index.php?id=education	Courses, events, glossary, clinical genomics guide app, quiz app	Health Professionals, academics, students, teachers
Genetics/Genomics Competency Center	https://genomicseducation.net/competency	Educational material, resources, competencies maps	Health Professionals
Center Genetics Education Canada–Knowledge Organization	https://geneticseducation.ca/	Educational resources, seminars, public resource, events	Health Professionals, Public
Australian Genomics Health Alliance	https://www.melbournebioinformatics.org.au/project/austgenomics/	Workshops	Health Professionals, students, academics
Centre for Genetics Education (CGE)	https://gardn.org.au/support-groups/directory/#!biz/id/594b367907ac801805955111	Online training modules, fact sheets, patient’s booklets, and pamphlets	Health Professionals, public
The Jackson Laboratory	https://www.jax.org/personalized-medicine/precision-medicine-and-you/genetics-vs-genomics	Online learning courses, training programs	Health Professionals, students, teachers, researchers
Genomic Applications in Practice and Prevention Network (GAPPNet™)	https://www.cdc.gov/genomics/translation/GAPPNet/index.htm/	General Genetic information, Fact sheets	Health Professionals
Genetics/Genomics Competency Center for Education (G2C2)	https://www.genomicseducation.net/	Online genomics educational materials	Health Professionals
National Library of Medicine: Genetics Home Reference	https://ghr.nlm.nih.gov/	Online material on basic genetics	Health Professionals, public
Omics Nursing Science & Education Network (ONSEN)—[National Human Genome Research Institute (NHGRI), National Cancer Institute (NCI), and National Institute of Nursing Research (NINR)]	https://omicsnursingnetwork.net/	Mentorship opportunities, pre- and/or post-doctoral training positions, research collaboration	Nurses
National Institute of Nursing Research -Summer Genetics Institute (SGI)	https://www.ninr.nih.gov/training/trainingopportunitiesintramural/summergeneticsinstitute	Summer course, lectures, and hands-on laboratory training	Nurses, academics, students
National Human Genome Research Institute: Talking Glossary	https://www.genome.gov/10002134/1999-release-talking-glossary	Online genetics glossary, text, audio and visual materials	Health Professionals, students, public
U.S. Surgeon General’s: My Family Health Portrait	https://phgkb.cdc.gov/FHH/html/index.html	Family History Resources	Public
National Cancer Institute: Cancer Genetics Risk Assessment and Counseling	https://www.cancer.gov/about-cancer/causes-prevention/genetics/risk-assessment-pdq	Genetic Education and Counseling	Health Professionals
Omics Nursing Science and Education Network (ONSEN)	https://omicsnursingnetwork.net/	Leveled knowledge matrix, list of mentors and list of pre- and post-doctoral training positions	Nurses, trainees, investigators, mentors
Beijing Genomics Institute	https://en.genomics.cn/	Youth science programs, videos, conference, events	Health Professionals, students, teachers, researchers
Genetic Database Kanehisa Laboratory in Kyoto University	https://www.genome.jp/en/about_dbget.html	Bioinformatics tools	Health Professionals, students, teachers, researchers
EuroGentest	http://www.eurogentest.org/index.php?id=894	Online courses, lessons, information on genetics	Public, Health professionals
Cold Spring Harbor Laboratory—DNA from the beginning	http://www.dnaftb.org/	Educational programs, courses	Public, students
